# The Relevance of a Diagnostic and Counseling Service for People Living With HTLV-1/2 in a Metropolis of the Brazilian Amazon

**DOI:** 10.3389/fpubh.2022.864861

**Published:** 2022-03-28

**Authors:** Felipe Teixeira Lopes, Renata Santos de Sousa, Jayanne L. Carvalho Gomes, Mariana Cayres Vallinoto, Aline Cecy Rocha de Lima, Sandra Souza Lima, Felipe Bonfim Freitas, Rosimar N. Martins Feitosa, Andrea Nazaré M. Rangel da Silva, Luiz Fernando A. Machado, Cintia Y. P. Aben-Athar, Eduardo Leitão Maia da Silva, Izaura M. V. Cayres Vallinoto, Antonio Carlos R. Vallinoto

**Affiliations:** ^1^Laboratory of Virology, Institute of Biological Sciences, Federal University of Pará, Belém, Brazil; ^2^Virology Section, Evandro Chagas Institute, Ananindeua, Brazil

**Keywords:** HTLV-1, HTLV-2, epidemiology, Belém, Amazonia

## Abstract

**Introduction:**

To identify the prevalence of infection in the urban area of the capital city of Belém, Brazil, the Laboratory of Virology of the Federal University of Pará implemented, as a public service, serological screening for human T-lymphotropic viruses 1 and 2 (HTLV-1/2) infection and, if necessary, counseling service and referral to specialized medical care. The project is funded by the National Council of Science and Technology, the Ministry of Health of Brazil and the Pan American Health Organization.

**Methods:**

From January 2020 to June 2021, 1,572 individuals of both sexes were approached to answer a questionnaire and were tested using an enzyme immunoassay (Murex HTLV-I+II, DiaSorin, Dartford, UK). Seropositive samples were confirmed as HTLV-1 and HTLV-2 infection by line immunoassay (INNO-LIA^®^ HTLV I/II Score, Fujirebio, Japan) and/or by real-time polymerase chain reaction. G and Fisher's exact tests were applied to identify the association between epidemiological characteristics and HTLV-1/2 infection.

**Results:**

Of the 1,572 screened individuals, 63.74% were females between the ages of 30 and 59 years (49.04%). Infection was confirmed in six individuals (0.38%), among whom three (0.19%) were infected with HTLV-1 and three with HTLV-2 (0.19%). Blood transfusion before 1993 was the main risk factor associated with the route of exposure to the virus (*p* = 0.0442). The infected individuals were referred to a counseling session with a nursing professional, and two patients who manifested signs and symptoms suggestive of myelopathy associated with HTLV were referred to a neurologist.

**Conclusion:**

The implementation of the screening service revealed the occurrence of moderate endemicity of HTLV-1/2 in Belém, reinforcing the importance of continuing the service as a means of establishing an early diagnosis and providing counseling as a measure to prevent and control viral transmission in the general population.

## Introduction

Human T-lymphotropic viruses 1 and 2 (HTLV-1 and HTLV-2) belong to the family *Retroviridae*, subfamily *Orthoretrovirinae*, and genus *Deltaretrovirus* (1), and both were isolated in the early 1980s (2, 3). HTLV-1/2 is characterized by the ability to cause a persistent and silent infection, and its forms of transmission are contact with contaminated organic fluids through sexual intercourse, syringe and needle sharing and breastfeeding (4, 5).

Although most individuals infected with the virus show no symptoms, HTLV-1 is associated with serious diseases, such as HTLV-1-associated myelopathy (HAM) and adult T-cell leukemia/lymphoma (ATL), pathologies that may occur in ~2–3% of people living with HTLV (PLHTLV) (2, 6, 7). HTLV-1 is also associated with other diseases, such as uveitis, infectious dermatitis, bronchitis, bronchiectasis, arthropathies, myositis, urinary dysfunction, and strongyloidiasis (8–10). For HTLV-2, there are reports that correlate it to cases of neurological disorders similar to HAM; however, there are no precise indications of the clinical manifestations of these disorders (11, 12).

The diagnosis of infection is initially performed using an enzyme-linked immunosorbent assay (ELISA) as a screening method for the detection of anti-HTLV-1/2 antibodies. Positive cases are referred for infection confirmation by Western blot, INNO-LIA and/or conventional (PCR) or real-time (qPCR) polymerase chain reaction assays (13). The first two tests are used for the detection of specific antibodies to different virus proteins, and PCR is used for the detection of proviral DNA, to differentiate between viral types and subtypes, and to quantify the proviral load (13–15).

Globally, it is estimated that 5 to 10 million individuals are infected with HTLV, with the distribution varying based on ethnic group, behavioral risk factors for infection and geographic location; southwestern Japan, sub-Saharan Africa and South America have the highest endemicity of the virus (16). In Brazil, there are an estimated 2.5 million PLHTLV (17), and Brazil is considered the country with the highest absolute number of HTLV-1/2 seropositive individuals in the world (18). The infection is endemic, being heterogeneously distributed among the different Brazilian regions and states, with Bahia, Maranhão and Pará recording the highest prevalence rates in Brazil (6, 17, 18).

Previous studies conducted in the state of Pará have reported high prevalence rates of HTLV infection in specific groups, such as blood donors (18, 19), pregnant women (20), indigenous populations (21), the Quilombola people (22) and people living with human immunodeficiency virus (PLHIV) (23). The prevalence of the virus in the metropolitan region of Belém, the most population-dense area in the state of Pará, is still unknown because of a lack of the early diagnosis of infection and diseases associated with HTLV (24), reinforcing the fact that the epidemiology of this viral agent should be better described for the capital of Pará.

It is of paramount importance the counseling of asymptomatic HTLV-1-infected subjects, to perform a clinical follow-up of the individual aiming to detect early the symptoms of diseases associated with the virus. Additionally, the lack of public policies for the active identification of cases of infection and subsequent counseling and screening for intrafamilial transmission results in a loss of productivity for those affected and their families. In addition, the absence of policies for preventing new infections and for providing adequate care for those living with HTLV-1 contributes to the increase in socioeconomic disparity between and within countries (25). Given this, the Laboratory of Virology of the Institute of Biological Sciences of Federal University of Pará (Universidade Federal do Pará—UFPA), with support from the Brazilian National Council for Scientific and Technological Development (CNPq, acronym in Portuguese) and the Pan American Health Organization (PAHO), implemented a public HTLV-1/2 serological screening and confirmation service for the general population of the city of Belém (metropolis of the Brazilian Amazon) and, if necessary, provided counseling and follow-up by a nurse and a referral for care by various health professionals (nurse, occupational therapist and doctor). In the present study, we aimed to describe the main results after the first year of service implementation.

## Methods

### Study Design

In the period from January 2020 to June 2021, the Laboratory of Virology of the Federal University of Pará began the HTLV-1/2 serological screening project in the city of Belém, the capital of the state of Pará, and tested a total of 1,572 individuals of both sexes who were categorized into the following age groups: children (7 to 11 years); adolescents (12 to 17 years); adults (18 to 59 years); and elderly individuals (>60 years).

The individuals were approached through an active search *via* the promotion of social actions in health, carried out primarily in community and religious centers in different neighborhoods in the capital. A portion of the sample consisted of people who voluntarily sought the Laboratory of Virology of UFPA, seeking a diagnosis for an infection, after extensive posts and dissemination on social networks and in a newspaper (printed and online) with high circulation in the state.

The participants were given basic information about HTLV and educated on the importance of diagnosing the infection and implementing prevention methods. All participants answered an epidemiological survey, after which peripheral venous blood was collected into 4-mL EDTA tubes. Subsequently, the samples were centrifuged to separate the plasma and cells (erythrocytes and leukocytes) and stored at −20°C until serological and molecular tests were performed at the UFPA Laboratory of Virology. The participants were informed that if they presented reactive or indeterminate results, blood would be collected again to confirm the infection and that if infection was confirmed, they would be referred to counseling and follow-up with a nurse. Individuals who manifested clinical signs suggestive of diseases associated with HTLV were referred to specialized medical care.

### Serological Screening

Plasma detection of total anti-HTLV-1/2 antibodies was performed using an ELISA (Murex HTLV-I+II, DiaSorin, Dartford, UK) following the manufacturer's protocol. Samples with reactive or indeterminate results (cut-off = 0.284) were subjected to confirmatory tests (INNO-LIA and/or qPCR).

### Line Immunoassay

LIA was used as a supplementary confirmation method for HTLV infection and used to differentiate viral types. An INNO-LIA^®^ HTLV I/II Score kit (Fujirebio, Japan) was used to detect anti-HTLV-1 and/or HTLV-2 antibodies following the manufacturer's protocol.

### DNA Extraction

For the analysis of HTLV proviral DNA, DNA was extracted from 200 μL of cell mass (leukocytes) with a QiaAmp DNA mini kit (Qiagen, Germany) following the manufacturer's protocol.

### Real-Time PCR

Samples with positive ELISA results that were subsequently confirmed by INNO-LIA were subjected to qPCR using the TaqMan system (Applied Biosystems, Foster City, CA) on the Applied Biosystems StepOne Plus Real Time PCR platform. The human albumin gene was used as an endogenous reaction control, and the viral gene regions *pol* (186 bp) of HTLV-1 and *tax* (75 bp) of HTLV-2 were used as viral confirmation and molecular differentiation targets (26). Each reaction contained 12.5 μL of TaqMan Universal PCR Master Mix (2X) (Applied Biosystems, Foster City, USA), 6.0 μL of ultrapure water, 0.5 μL of each primer, 0.5 μL of each probe and 5.0 μL of DNA, resulting in a total volume of 25 μL. The following temperature cycles were used: 95 °C for 10 mins, followed by 45 cycles of 95 °C for 15 secs and 60°C for 1 min for primer and probe binding.

The following primers were used in the reactions: 5′-CCCTACAATCCAACCAGCTCAG-3′ (HTLV-1F), 5′-GTGGTGAAGCTGCCATCGGGTTTT-3′ (HTLV-1R), 5′-CGATTGTGTACAGGCCGATTG-3′ (HTLV-2F), 5′-CAGGAGGGCATGTCGATGTAG**-**3′ (HTLV-2R), 5′-GCTGTCATCTCTTGTGGGCTGT-3′ (Albumin F), and 5′-AAACTCATGGGAGCTGCTGGTT-3′ (Albumin R). The probe sequences were as follows: FAM-5′- CTTTACTGACAAACCCGACCTACCCATGGA-3′-MGB (HTLV-1), FAM-5′- TGTCCCGTCTCAGGTGGTCTATGTTCCA-3′-MGB (HLTV-2) and FAM-5′- CCTGTCATGCCCACACAAATCTC-3′-MGB (Albumin) (26).

### Statistical Analysis

The data obtained from the epidemiological survey answered by the participants was input in a database in Epi-Info 7.2. Statistical analyses were performed using BioEstat 5.3 (27) and GraphPad Prism 6.0. The studied variables were analyzed using descriptive statistics, and the estimated prevalence was analyzed by point and confidence interval (95% confidence interval [CI]) estimators. To identify the epidemiological characteristics associated with HTLV infection, the chi-square and Fisher's exact tests were applied, adopting a significance level of 95% (*p* < 0.05).

### Ethical Aspects

This study complied with ethical precepts and was submitted to and approved by the Human Research Ethics Committee of the Health Sciences Institute of UFPA and by the National Research Ethics Committee (CAAE No. 27290619.2.0000.0018; opinion: 4.351.470), in accordance with Resolution no. 466/12 of the Ministry of Health of Brazil. Individuals over 18 years of age who agreed to participate signed an informed consent form. Children under 18 years of age signed an informed assent form, and their legal representatives signed an informed consent form, authorizing their participation in the study.

## Results

### Serological Screening and Infection Confirmation

Of the 1,572 individuals evaluated, 63.74% were female, with a mean age of 41 years, ranging from 30 to 59 years (49.04%). Among the other characteristics identified in the epidemiological survey, 54.03% self-reported being mixed race, 33.81% reported having a higher education, and 38.69% reported having a family income equal to or greater than five times the minimum wage, with no significant differences between infected and non-infected individuals (Table 1).

**Table 1:** General characteristics of the study population.

**General characteristics**	**Study population**			***p***
	**Total** ***n* (%)**	**Positive** ***n* (%)**	**Negative** ***n* (%)**	
**Prevalence**	1,572 (100)	6 (0.38)	1,566 (99.62)	
**Sex**				
Male	570 (36.26)	2 (33.33)	568 (36.27)	0.7845
Female	1,002 (63.74)	4 (66.67)	998 (63.73)	
**Ethnicity**				
White	459 (29.59)	2 (33.33)	457 (29.58)	0.7476
Black	223 (14.38)	2 (33.33)	221 (14.30)	
Mixed	838 (54.03)	2 (33.33)	836 (54.11)	
Asian	31 (2.00)	0	31 (2.01)	
**Age group (years)**				
7 to 11	28 (1.84)	0	28 (1.84)	0.1107
12 to 17	68 (4.46)	0	68 (4.48)	
18 to 29	382 (25.06)	0	382 (25.15)	
30 to 59	748 (49.04)	1 (16.67)	747 (49.18)	
≥ 60	299 (19.60)	5 (83.33)	294 (19.35)	
**Education level**				
Illiterate	6 (0.38)	0	6 (0.38)	0.3691
Primary	162 (10.35)	4 (66.66)	158 (10.14)	
Secondary	423 (27.03)	1 (16.67)	422 (27.07)	
Higher	529 (33.81)	1 (16.67)	528 (33.87)	
Graduate	445 (28.43)	0	445 (28.54)	
**Income (minimum wages)**				
<1	105 (6.97)	1 (16.67)	104 (6.93)	0.8907
1 to 2	470(31.19)	2 (33.33)	468 (31.18)	
3 to 4	349 (23.15)	1 (16.67)	348 (23.18)	
≧5	583 (38.69)	2 (33.33)	581 (38.71)	

After obtaining the serology results for anti-HTLV-1/2 antibodies, six individuals were positive and were contacted by telephone to schedule an appointment for blood collection to confirm the viral infection. The new samples were subjected to the same testing sequence, and ELISA and INNO-LIA were performed again. Additionally, qPCR was performed as previously described. Infection was confirmed in six individuals (0.38%; CI: 0.07–0.69%), with 3 (0.19%) infected by HTLV-1 and 3 infected by HTLV-2 (0.19%). Among those infected, 66.7% were women aged 49 to 87 years (Table 2).

**Table 2:** Results of tests performed for the screening and confirmation of HTLV infection.

**Patient**	**Age**	**Sex**	**ELISA**	**INNO-LIA**								**qPCR**		**Result**
			**Anti-HTLV 1/2**	**gag p19 I/II**	**gag p24 I/II**	**env gp46 I/II**	**env gp21 I/II**	**gag p19-I**	**env gp46-I**	**env gp46-II**	**Profile**	***pol-I***	***tax-II***	
#01	67	M	Reactive	+	–	+	+	+	+	–	HTLV-1	ND	ND	HTLV-1
#02	73	F	Reactive	+	–	+	+	–	–	–	HTLV	Detectable	ND	HTLV-1
#03	60	F	Reactive	+	+	+	+	+	+	–	HTLV-1	Detectable	ND	HTLV-1
#04	49	F	Reactive	+	+	+	+	–	–	+	HTLV-2	ND	Detectable	HTLV-2
#05	61	M	Reactive	–	–	+	+	–	–	–	HTLV	ND	Detectable	HTLV-2
#06	87	F	Reactive	+	+	+	+	–	–	+	HTLV-2	ND	Detectable	HTLV-2

^*^ ND, Not detectable; ^*^+, Positive; ^*^-, Negative; ^*^M, Male; ^*^F, Female.

INNO-LIA indicated two cases of an HTLV profile without defining the viral type, and qPCR confirmed the presence of proviral DNA of HTLV-1 and HTLV-2. One HTLV-1-positive individual, as determined by INNO-LIA, did not have proviral DNA detected by qPCR (Table 2).

### Clinical and Epidemiological Characteristics of Individuals Seropositive for HTLV-1/2

When evaluating the responses obtained in the epidemiological survey answered by the six HTLV carriers, blood transfusion was the main risk factor associated with the virus exposure route (p = 0.0442) (Table 3). However, when this result was stratified into “before” and “after” 1993, the year in which screening for HTLV in blood bags became mandatory in blood centers in Brazil, statistical significance was not maintained (Table 3).

**Table 3:** Risk factors associated with HTLV infection.

Risk factors^*^	**HTLV-1/2**			*p* ^**^
	**Total** **(%)**	**Positive** **(%)**	**Negative** **(%)**	
**Tattoo**				
Yes	273 (17.65)	1 (20.00)	272 (17.64)	0.6302
No	1,274 (82.35)	4 (80.00)	1,270 (82.36)	
Not reported	25	1	24	
**Piercing**				
Yes	105 (06.92)	0	105 (06.94)	0.7977
No	1,413 (93.08)	5 (100)	1,408 (93.06)	
Not reported	54	1	53	
**Blood transfusion**				
Yes	109 (07.18)	2 (40.00)	107 (07.07)	0.0442^***^
No	1,410 (92.82)	3 (60.00)	1,407 (92.93)	
Not reported	53	1	52	
**Blood transfusion period**				
Before 1993	26 (27.08)	2 (100)	24 (25.53)	0.0713
After 1993	70 (72.92)	0	70 (74.47)	
**Use of illicit drugs**				
Yes	90 (06.02)	0	90 (06.04)	0.7310
No	1,404 (93.98)	5 (100)	1,399 (93.96)	
Not reported	78	1	77	
**Sexually active**				
Yes	1,303 (87.86)	5 (83.33)	1,298 (87.88)	0.5406
No	180 (12.14)	1 (16.67)	179 (12.12)	
Not reported	89	0	89	
**Use of condoms**				
Yes	585	1 (20.00)	584 (43.52)	0.1705
No	532	4 (80.00)	528 (39.34)	
Sometimes	230	0	230 (17.14)	
Not reported	225	1	224	
**Practices sex for money**				
Yes	20	0	20 (01.40)	0.2817
No	1,416	5 (100)	1,411 (98.60)	
Not reported	136	1	135	
**Diagnosis of STI**				
Yes	104	1 (20.00)	103 (07.24)	0.5765
No	1,223	3 (60.00)	1,220 (85.79)	
Does not know	100	1 (20.00)	99 (06.96)	
Not reported	145	1	144	

* *The relative frequencies only of the answered questions are shown*.

** *Unanswered questions were not considered in the statistical analysis*.

*** *Significant p value*.

The infected individuals received their respective laboratory diagnostic for the infection and were referred for a first counseling session with a nurse who took the medical history of the patients and talks about some virus' aspects, such as transmission modes, diseases associated, intrafamilial segregation, prevention, and treatment. The close relatives (parents, spouses and children) of the six patients were invited to undergo testing for possible intrafamilial transmission; however, to date, they have not yet responded to our first call.

Of the six individuals with confirmed cases, four were asymptomatic. Two female HTLV-2-positive individuals had clinical signs suggestive of neurological disorders and were referred for evaluations by a neurologist, as described below.

Patient **#04** (HTLV-2), female, single, 49 years old, had incomplete primary education and reported in the survey that she had been pregnant three times and breastfed for more than 6 months. At the time of the interview, she reported not having an active sex life. During counseling with a nurse, she reported feeling hand tremors, urinary urgency, swelling and joint pain, with restricted movements and gait changes.

Patient **#06**, widower, 87 years old, was hospitalized during the study with severe pulmonary fibrosis and presented with alternating consciousness, which prevented a complete neurological evaluation. It was possible to observe only the absence of clear signs of HAM, but there was a history of urinary incontinence associated with motor deficit (leg weakness) but without signs suggestive of a pyramidal corticospinal tract injury.

## Discussion

Investigations of the prevalence of HTLV-1/2 infection in the state of Pará have been conducted in specific population groups, such as blood donors, indigenous populations, the Quilombola people, PLHIV and Japanese immigrants (22, 28–30). The present study provides seroepidemiological information on the prevalence of HTLV-1 and HTLV-2 in the general population of Belém, the capital of the state of Pará.

A study by Silva et al. (24), conducted between 2014 and 2015, showed a 2% prevalence of HTLV infection (1.4% for HTLV-1 and 0.5% for HTLV-2) in a sample from the city of Belém, classifying this location as an area of moderate endemicity. The prevalence found in the present study of 0.38% was well below that described by Silva et al. (24); this difference may be due to (i) a possible reduction in prevalence over the 6 years separating the studies; (ii) the fact that Silva et al. (24) sampled adult individuals who were passing public places such as the Ver-o-Peso Complex, a location with a high circulation of people from other municipalities of the metropolitan region of Belém, which differs from the present study, which evaluated only people residing in Belém; (iii) sample size; or (iv) differences in the sensitivity of the different ELISAs used in the two studies. Currently, we are collecting seroepidemiological information from neighboring municipalities that compose the metropolitan region of Belém, for example, Ananindeua, Marituba and Benevides, with the objective of separately evaluating prevalence and thus identifying the overall occurrence of infection in the region. Our results did not differ from those observed by Maneschy et al. (31) in a study conducted between 2015 and 2019 in a blood center in the state of Pará; the results indicated an HTLV prevalence of 0.3% (1476/453,626) among blood donors, among whom most positive individuals were from the metropolitan region of Belém. In another study conducted with pregnant women, the estimated prevalence of HTLV was 0.6% (32).

During the screening and confirmation analyses, two individuals were identified by INNO-LIA with a serological profile of HTLV, and qPCR molecular analysis confirmed the presence of HTLV-1 proviral DNA in one individual and HTLV-2 proviral DNA the other. Another individual was positive for HTLV-1 based on INNO-LIA diagnostic criteria, but proviral DNA was not detected by qPCR. These serological (INNO-LIA) and molecular (qPCR) methods have been used and endorsed for the confirmatory and differential diagnosis of HTLV-1 and HTLV-2 infections (13). Differences in results between serological and molecular methods are frequent and result from differences in the sensitivity and specificity of the tests (26). Furthermore, Caterino-de-Araújo and Rodrigues Campos (33) described difficulties in the laboratory diagnosis of the infection, including the presence of incomplete HTLV viral particles (characterized by the absence of a viral gene, such as the *tax* gene), low quality of extracted DNA, mutations and a low proviral load, which can potentially lead to the absence of gene amplification and thus result in a false-negative diagnosis for infection. Therefore, the use of a single laboratory method to confirm and differentiate HTLV-1/2 infection is not recommended; it is necessary to adopt complementary confirmatory assays to minimize diagnostic and prevalence estimate errors.

In addition to an accurate laboratory diagnosis that allows identifying the prevalence of infection in a given geographic area, it is necessary to implement a counseling and follow-up service for PLHTLV to provide efficient clinical, laboratory and psychological monitoring. Rosadas et al. (34) emphasize the importance of including the multiprofessional screening of HTLV in the routine care provided in services for people infected with HIV and other STIs. For this reason, all individuals diagnosed in our study were referred to initial counseling with a nurse, at which time they were informed about HTLV, its modes of transmission and prevention, associated diseases and supportive therapies for infection-related symptoms. Subsequently, those individuals who, in counseling, complained of neurological changes were referred to a neurologist. Of the six individuals with confirmed cases of infection, four individuals were asymptomatic. Interestingly, the two individuals who presented complaints suggestive of neurological disorders were infected with HTLV-2. Although there is some rare evidence in the literature associating this viral type with neurological diseases similar to HAM (11, 12), the initial evaluation by the neurologist did not identify the presence of clinical changes that confirmed a diagnosis of HAM.

## Conclusions

The results presented here are part of the first year of a screening, confirmation, counseling and multiprofessional follow-up service for PLHTLV offered through the Laboratory of Virology of the Federal University of Pará and implemented as part of a National Network of laboratories, funded by the Ministry of Health and PAHO. The main limitation of the study can be considered the difficulty of convincing the seropositive individual to perform a new blood collection in order to carry out the confirmatory tests. On the other hand, the main strength of the study is to contribute with the prevention and control measures for HTLV infection to reduce transmission and to identify the main risk factors associated with infection, characterizing the individuals or groups that may be more vulnerable to HTLV, and thus provide information that will support the development of public health policy strategies to combat the spread of this virus in Brazil.

## Data Availability Statement

The raw data supporting the conclusions of this article will be made available by the authors, without undue reservation.

## Ethics Statement

The studies involving human participants were reviewed and approved by Human Research Ethics Committee of the Health Sciences Institute of the Federal University of Pará and by the National Research Ethics Committee (CAAE No. 27290619.2.0000.0018). The patients/participants provided their written informed consent to participate in this study.

## Author Contributions

AV and IC conceived and designed the study. FL, MV, AL, and FF performed the laboratory experiments. FL, RS, JC, MV, AL, RF, AR, and LM performed the fieldwork. CA-A and EM performed clinical follow-ups. FL and AV wrote the manuscript. All authors read and approved the final version of the manuscript.

## Funding

This study received financial support from the National Council of Science and Technology (CNPq # 442522/2019-3 and CNPQ # 302935/2021-5) and the Ministry of Health of Brazil and the Pan American Health Organization (#SCON2021-00310).

## Conflict of Interest

The authors declare that the research was conducted in the absence of any commercial or financial relationships that could be construed as a potential conflict of interest.

## Publisher's Note

All claims expressed in this article are solely those of the authors and do not necessarily represent those of their affiliated organizations, or those of the publisher, the editors and the reviewers. Any product that may be evaluated in this article, or claim that may be made by its manufacturer, is not guaranteed or endorsed by the publisher.
